# High levels of RNA-editing site conservation amongst 15 laboratory mouse strains

**DOI:** 10.1186/gb-2012-13-4-r26

**Published:** 2012-04-23

**Authors:** Petr Danecek, Christoffer Nellåker, Rebecca E McIntyre, Jorge E Buendia-Buendia, Suzannah Bumpstead, Chris P Ponting, Jonathan Flint, Richard Durbin, Thomas M Keane, David J Adams

**Affiliations:** 1 Wellcome Trust Sanger Institute, Hinxton, Cambridge, CB10 1HH, UK; 2 MRC Functional Genomics Unit, Department of Physiology, Anatomy and Genetics, University of Oxford, South Parks Road, Oxford, OX1 3QX, UK; 3 The Wellcome Trust Centre for Human Genetics, Roosevelt Drive, Oxford OX3 7BN, UK

## Abstract

**Background:**

Adenosine-to-inosine (A-to-I) editing is a site-selective post-transcriptional alteration of double-stranded RNA by ADAR deaminases that is crucial for homeostasis and development. Recently the Mouse Genomes Project generated genome sequences for 17 laboratory mouse strains and rich catalogues of variants. We also generated RNA-seq data from whole brain RNA from 15 of the sequenced strains.

**Results:**

Here we present a computational approach that takes an initial set of transcriptome/genome mismatch sites and filters these calls taking into account systematic biases in alignment, single nucleotide variant calling, and sequencing depth to identify RNA editing sites with high accuracy. We applied this approach to our panel of mouse strain transcriptomes identifying 7,389 editing sites with an estimated false-discovery rate of between 2.9 and 10.5%. The overwhelming majority of these edits were of the A-to-I type, with less than 2.4% not of this class, and only three of these edits could not be explained as alignment artifacts. We validated 24 novel RNA editing sites in coding sequence, including two non-synonymous edits in the *Cacna1d *gene that fell into the IQ domain portion of the Cav1.2 voltage-gated calcium channel, indicating a potential role for editing in the generation of transcript diversity.

**Conclusions:**

We show that despite over two million years of evolutionary divergence, the sites edited and the level of editing at each site is remarkably consistent across the 15 strains. In the *Cds2 *gene we find evidence for RNA editing acting to preserve the ancestral transcript sequence despite genomic sequence divergence.

## Background

The adenosine deaminase acting on RNA (ADAR) family of enzymes is capable of modifying adenosine residues to inosines [1]. Inosine is interpreted by the transcriptional machinery as guanosine, and thus appear as A-to-G mismatches in cDNA sequences. The ADARs bind to double-stranded regions of RNA and can modify multiple neighboring adenosines until a tolerance level is reached and the structure of the RNA becomes destabilized [2]. RNA editing in coding regions can lead to alteration in protein function and increased transcript diversity. The best known example of this is editing of neural serotonin receptor *HTR2C *gene transcripts, which are edited at five sites in close proximity to each other [3], thereby producing a diverse repertoire of 28 mRNAs and 20 protein isoforms [4]. Isoform composition and editing frequency are lower during early developmental stages and gradually increase with age until adulthood [3]. RNA editing can also induce alternative splicing; for example, the enzyme ADARB1 edits its own pre-mRNA, which leads to expression of a protein with diminished catalytic activity [5]. The functional consequence of RNA editing of non-coding sequence is not well understood, although it has been suggested that editing of UTR sequences may be associated with their nuclear retention [6]. RNA editing occurs in all metazoans, but the level of editing differs substantially across species. It has been estimated that one nucleotide in every 17,000 is edited in the rat brain transcriptome [7], while in the human brain transcriptome the number of RNA editing sites is thought to be an order of magnitude higher [8,9].

Large-scale efforts to generate cDNA sequences, and to annotate reference genomes, have greatly facilitated the discovery of RNA editing sites in the transcriptome of several species. Zaranek *et al*. [9] curated several tens of gigabases of human and mouse cDNA sequence to derive one of the most comprehensive surveys of RNA editing to date. More recently, the advent of second-generation sequencing technologies, and the development of the RNA-seq method, has made it possible to sequence the entire transcriptome [10]. Recently, several authors have attempted to use RNA-seq data to discover RNA editing sites. Li *et al*. [11] predicted approximately 10,000 RNA editing sites from RNA-seq data from human B-cells and reported all 12 possible base changes. Bahn *et al*. [12] used a different computational methodology to report a similar number of RNA editing sites in the transcriptome of a human glioblastoma cell line but found that the vast majority of edits (approximately 70%) were of the A-to-I type. It is clear from these discordant results that the accurate identification of true RNA editing sites is computationally challenging and great care must be taken to identify and remove artifacts such as alignment errors.

In this paper, we present the first comprehensive survey of RNA editing in the mouse genome across a diverse set of 15 mouse strains that together represent all of the major laboratory mouse lineages. To achieve this, we start with detailed knowledge of the genomic variation across the strains [13] and combined this information with single nucleotide variant (SNVs) called from RNA-seq data to generate an initial set of candidate edit sites, which were then systematically filtered to produce a highly accurate set of RNA editing sites that was dominated by A-to-I edits (Additional file 1). Using this filtered set of RNA editing sites, we investigate the pattern of RNA editing and used a knowledge-driven approach to significantly improve the sensitivity of the catalogue, especially in regions where there are clusters of RNA edits within the transcriptome. Our analysis produces one of the most comprehensive pictures of the extent of RNA editing in a vertebrate genome with an estimated false discovery rate (FDR) of between 2.9 and 10.5%. While most RNA editing sites lie outside of protein coding regions, we identified non-synonymous edits that contribute to transcript diversity and possibly protein diversity. Our analysis suggests that RNA editing in protein coding sequence is more commonly found in receptor and ion transport functional classes.

## Results

### Identification of cDNA/genome mis-match sites

**Figure 1:**
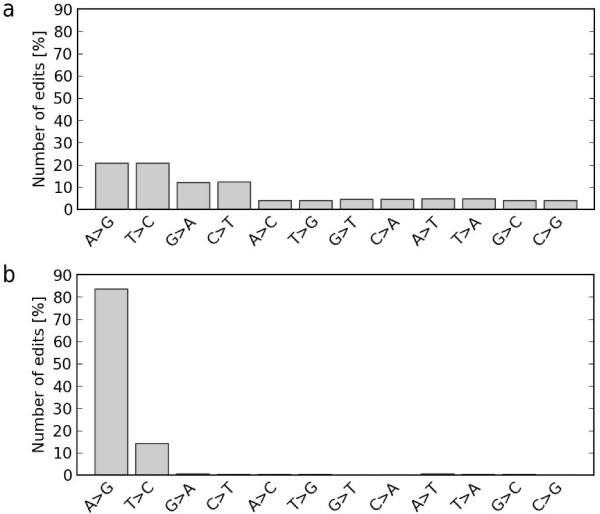
**Editing pattern of **(a) **the initial raw candidate set and **(b) **the final filtered set**.

RNA was extracted from the brains of mice from 15 strains (129S1/SvImJ, C57BL/6NJ, C3H/HeJ, A/J, AKR/J, DBA/2J, LP/J, CBA/J, BALB/cJ, NZO/HlLtJ, NOD/ShiLtJ, CAST/EiJ, PWK/PhJ, WSB/EiJ and SPRET/EiJ). cDNA was then generated and sequenced [ENA:ERP000614], and the resulting reads (paired end 76 bp reads) were aligned to the mouse reference sequence (mm9) with BWA [14]. At least two biological replicates were generated for each strain. SNVs were called using samtools/bcftools [15] as described in the Materials and methods. The initial set of variant calls consisted of 304,817 candidate RNA editing sites and contained a large proportion of non A-to-G mismatches (Figure 1a). Manual inspection of these calls revealed that the vast majority of these sites could be attributed to the misalignment of short read sequences (Figures s1 to s3 in Additional file 2) primarily due to the placement of reads to multiple sites and mis-alignments around splice site junctions.

The reads localized to candidate RNA editing sites were then realigned in a splice-aware manner using exonerate [16] and all sites with reads that were not uniquely mapped were removed, resulting in a smaller set of candidate editing sites totaling 98,061 positions. Additional filters were then used that required a minimum cumulative depth of 10 reads per strain, and sites where we observed any mismatch in the genomic reads at a position were removed. To avoid artifacts from sequencing errors or possible low-level contamination, we also required an RNA editing site to be observed in at least two biological replicates of a strain. In addition, we discarded sites where reads with mismatches were biased in orientation (Figure s2 in Additional file 2). As indicated above, the major source of systematic error stemmed from the mis-alignment of reads spanning splice sites. Two filters, End Distance Bias and, to a greater extent, Variant Distance Bias, which evaluate biases in the position of mismatches within reads, proved extremely effective at removing these artifacts (Materials and methods; Figures s3 and s4 in Additional file 2). Following the application of these filters the set of RNA editing sites was composed of 5,579 positions, of which only 2.4% were non A-to-G or T-to-C mismatches (Figure 1b). Figure s5 in Additional file 2 shows the filtering steps that we applied and the effect of each filter on the number and type of RNA editing sites in the dataset.

### Call set validation

We selected a random set of 611 calls from both the filtered set of 5,579 RNA editing sites and the much larger set of unfiltered candidates and validated these on the Sequenom platform [17] by genotyping genomic DNA from C57BL/6NJ and cDNA from each of the sequenced strains (Table s1 in Additional file 3). Approximately 30% of the assays yielded no signal or a signal that was inconsistent with the genomic DNA control and were excluded from further evaluation. Most of the failed assays appeared to be the result of Sequenom signals derived from multiple loci or a mixed PCR product. Comparison of the results from Sequenom assays to the calls made from the RNA-seq data revealed a discrepancy of 10.5%. Since this value was calculated as the number of sites that were not confirmed by Sequenom genotyping divided by the sum of the unconfirmed and confirmed sites, the figure of 10.5% is likely to reflect the upper boundary of the true FDR. Furthermore, a significantly higher proportion of sites where the editing level was less than 20% of transcripts did not validate by Sequenom genotyping. After manual inspection of these sites in the RNA-seq alignments we find no obvious systematic calling bias, suggesting that the sensitivity and specificity of Sequenom genotyping is, in these cases, lower than that of RNA-seq.

Further manual inspection of the non A-to-G edits in the refined call set revealed that the T-to-C mismatches were largely the result of uncertainties in the assignment of transcripts to a strand on the mouse assembly, or were the result of calls made in antisense transcripts. For example, the cluster of A-to-G mismatches (forward strand) that overlap the *Zscan30 *gene (reverse strand) appear as a novel form of T-to-C editing. However, the presence of another gene, *Zfp397*, and the *Rpl19-ps7 *pseudogene on the forward strand just upstream of *Zscan30 *suggests that antisense transcription or missing annotation are more likely explanations for the editing pattern at this site (Figure s6 in Additional file 2). The vast majority of the other non A-to-G or T-to-C mismatches could be explained as obvious alignment artifacts or low-quality mapping regions. Nineteen of the non A-to-G editing sites were amplified from cDNA and subjected to Sequenom genotyping. All were confirmed as false positives. Thus, based on this result we find that virtually all non A-to-G or non T-to-C mismatches are false positives. Assuming that the error rate for all types of mismatches are comparable, we estimated that the FDR of our call set is 2.9% (calculated using the formula k × s/N, where k is number of non-A-to-G and non-T-to-C calls and N is the number of all calls. The factor s = 12/10 increases the FDR estimate in order to account for errors in A-to-G and T-to-C bins). Interestingly, computational analysis followed by manual inspection of our final call set revealed only three non A-to-G edits that could not be explained as alignment artifacts, including a non-synonymous coding C-to-T mismatch in the *Mfn1 *gene at chr3:32,460,397 (Figure s7 in Additional file 2), which was validated by Sanger sequencing (Figure s8 in Additional file 2) and is compatible with the known C-to-U form of editing.

### Knowledge-driven extension of the filtered set

As the calling procedure made no assumptions about the nature of editing, we were able to show that adenosine deamination is the primary form of RNA editing in mouse. In order to obtain a high-confidence set of calls, our initial calling filters were quite strict and possibly removed many true edits. Having established that most of the single-nucleotide changes in the transcriptome are driven by ADARs, we extended the set by taking advantage of the known property of ADAR editing that often modifies multiple bases in close proximity to each other. Any mismatches within 10 bp from an RNA editing site were iteratively added to the pool of calls along with clusters of eight or more mismatches of the same type within a window of 20 bp. All of the variants identified using this approach were A-to-G substitutions, and the number of RNA editing sites was increased to 7,389 (Figure s5 in Additional file 2). We tested 23 of these clustered edits and all were confirmed by Sanger sequencing (Materials and methods; Figure s9 in Additional file 2).

We next compared our extended set of 7,389 RNA editing sites and our initial candidate set of calls (304,817 sites) to RNA editing sites predicted in previous studies [9,18,19]. We found sequence coverage for 38% (313) of the sites predicted by Neeman *et al*. [18] and called 23% of these sites in our extended call set. When we compared our calls to the set described by Rosenberg *et al*. [19], 28 sites were covered by sequence reads in our study but none of these sites were present in the initial set of candidate RNA editing calls we produced. Manual inspection of all 28 positions in our mouse transcriptome data failed to provide support for 24 positions as being subject to RNA editing. Of the four remaining, one position at chr15:99,239,051 shows clear evidence of RNA editing but was inadvertently filtered out of the extended call set because of a random base mismatch in genomic sequence at this position. There were also three other sites (chr2:121,978,638, chr3:119,135,669 and chr3:144,259,976) where occasional C-to-T mismatches were visible, but these occurred with a frequency that was too low to make a confident call that RNA editing was operative at these positions. Another set we examined was from the study of Zaranek *et al*. [9]. We found an extremely low overlap of our calls with this set: only 5 out of 1,528 sites (0.3%) were confirmed by our study. Interestingly, 62% (944) of the sites in the Zaranek *et al*. study were found as SNPs in one of the 17 strains we sequenced, suggesting that these calls are heavily contaminated with genomic variants.

### Editing rate and the sensitivity of variant calling

**Figure 2:**
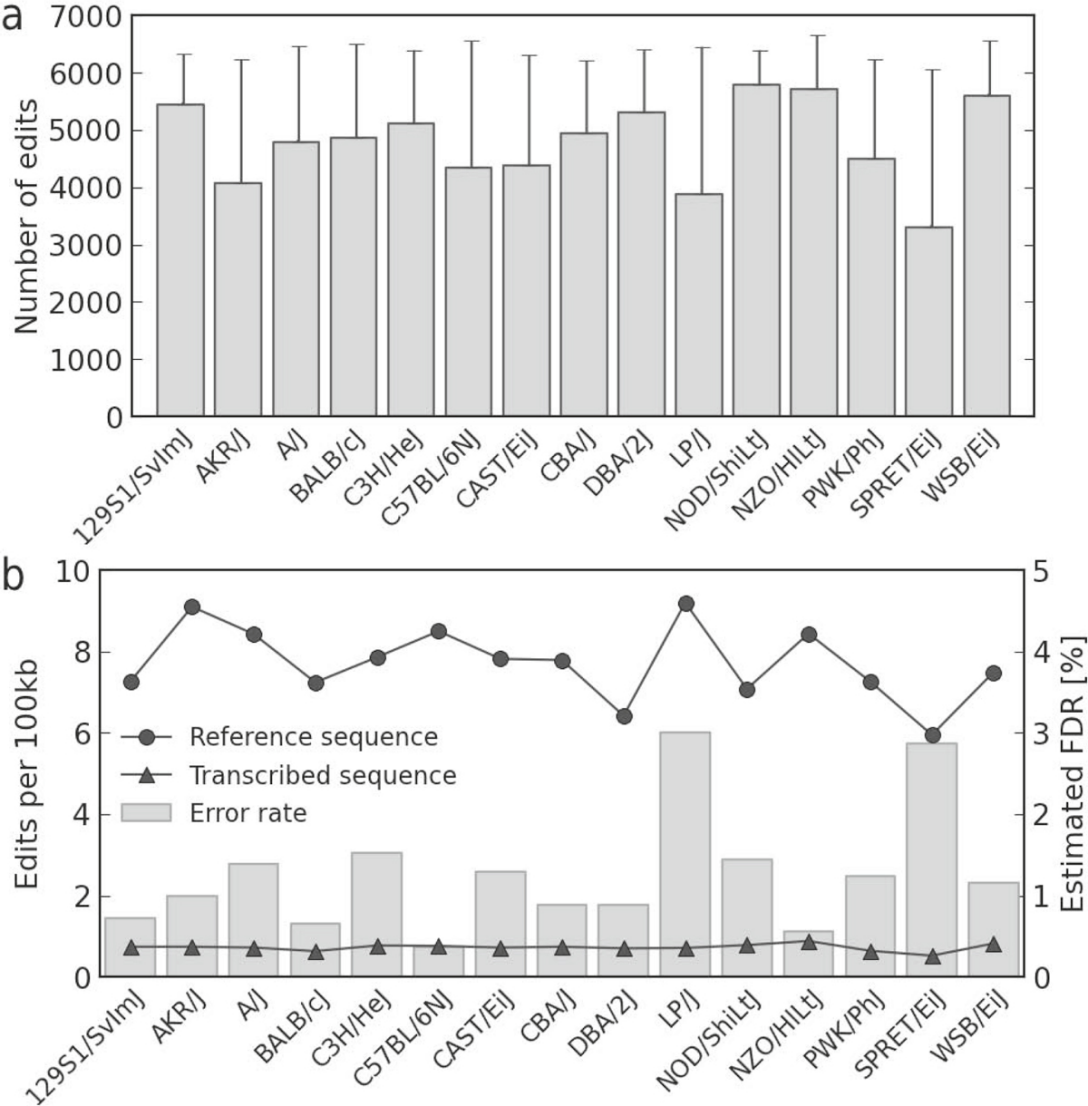
**The extent of RNA editing in individual mouse strains**. **(a) **Total number of edits per strain with error bars indicating the low-depth correction as explained in the text. **(b) **The editing rate is shown as the number of edited bases per 100 kbp of genomic reference sequence and as the number of inosines in 100 kbp of transcribed sequence. The error rate is calculated as the proportion of non A-to-G mismatches per strain.

We observed substantial variation in the number of predicted editing sites across the 15 strains, with an average of 4,800 edits per strain (standard deviation 700) being called (Figure 2a). We reasoned that a certain proportion of this variability could be attributed to differences in the sequence depth of each transcriptome rather than biological differences between strains. Indeed, we found that for only 21% of RNA editing sites can we be certain that an edit was not missed due to low depth in one of the strains at a 5% significance level of a one-sided binomial test (Materials and methods). Using a 95% level of confidence that RNA editing was not missed due to low sequence depth, the average number of RNA editing sites per strain increased to 6,383 and the standard deviation decreased to 153.

In our extended set, we observed six to ten RNA editing sites per 100 kb of accessible reference sequence, or less than one inosine per 100 kb of transcribed sequence (Figure 2b). However, we suspected that the true editing rate may be somewhat higher as manual inspection of 28 editing clusters revealed that due to very low levels of editing, we may have missed around half of edited positions from our call set, particularly when these sites were tightly clustered. Nonetheless, based on this evaluation, missing sites account for just 4% of the total inosines owing to the fact that the editing levels at these positions is very low. These sites are extremely challenging to call computationally as they are difficult to distinguish from random sequencing errors. Higher sequencing depth would help provide support for these positions as sites of RNA editing.

### Sequence context

Most RNA editing occurred in clusters of two or more sites (5,233 sites in 1,582 clusters) but 30% (2,156) of edits occurred at single sites (although this may, in part, just reflect our sensitivity to detect edited positions, as described above). Our analysis of local sequence motifs showed a distinct trend towards the UAG motif found by others [12,20]. On the other hand, Higuchi *et al*. [21] experimentally showed that ADARs require a duplex structure that does not depend on the sequence surrounding the edited position and we find that 25% of RNA editing sites have neither a T immediately 5' nor a G immediately 3' of the edited base. Interestingly, the highest editing levels were observed at UAU and UAG triplets (Figure s10 in Additional file 2). In humans, UAU sites have been reported as the fifth most frequently edited sequence context, with UAG predominating [22]. The differences we observe in mouse may reflect true biological differences between mouse and human ADARs, or differences in the RNA editing targets between species. The improved resolution we gained by performing our analysis using sequence contexts derived from 18-fold more RNA editing events than were used for the analysis in the abovementioned human study may also explain the differences we report.

### RNA editing levels

**Figure 3:**
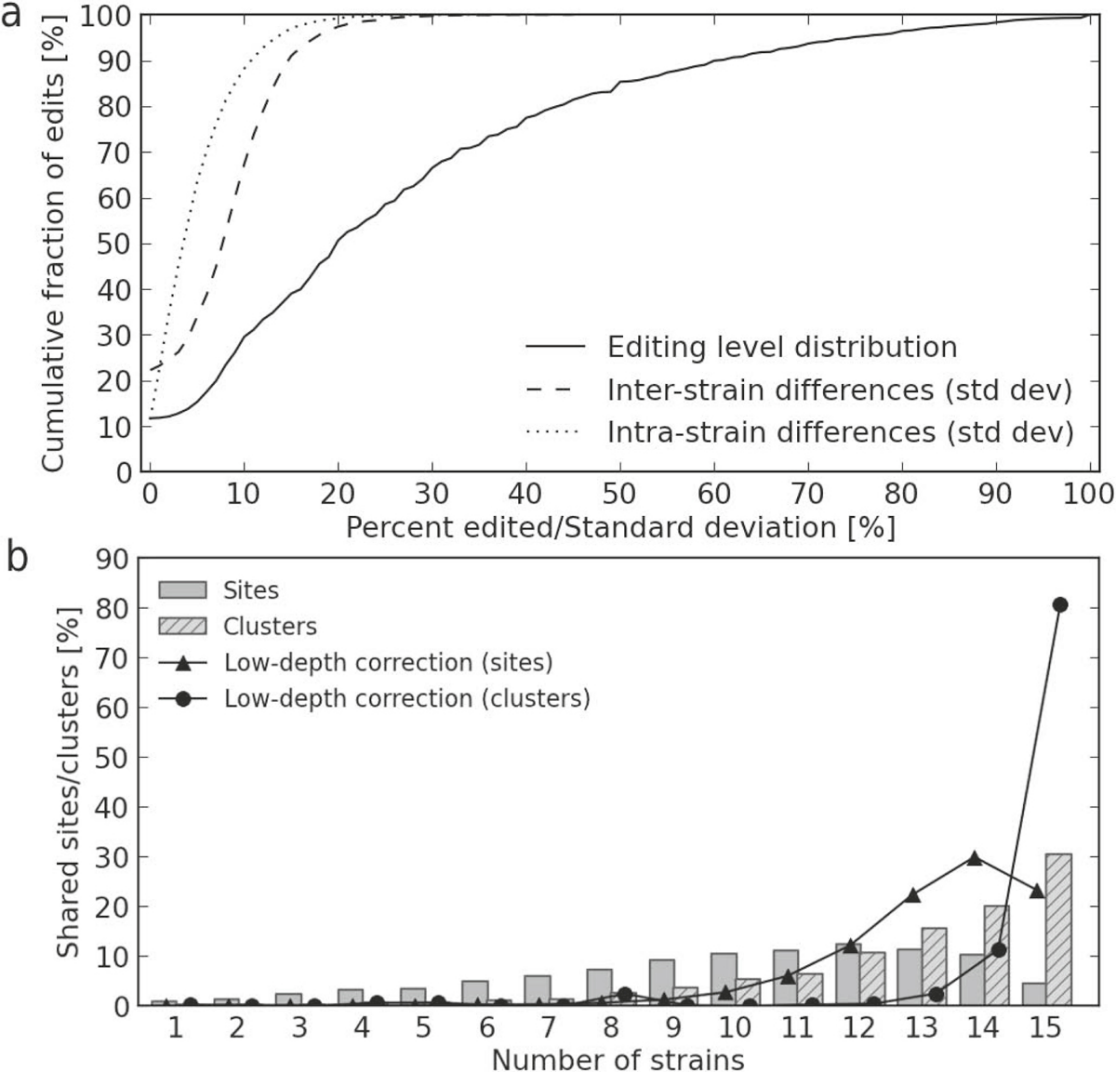
**RNA editing sites characterized by the level of editing and their presence in multiple mouse strains**. **(a) **The overall level of editing is determined by the number of reads with/without the edited base per site and is shown over all sites/strains (solid line). The variability in the level of editing is shown as the distribution of standard deviations (std dev) from average values at each site both within (dashed line) and across strains (dotted line). For example, 90% of all sites have 60% or fewer reads edited and the standard deviation for 90% of all sites is less than 10 to 15%. **(b) **The frequency of sharing of sites (solid bars) and clusters across the strains (dashed bars). The lines show the hypothetical number of shared edits/clusters when uneven coverage and expression are taken into account, as explained in text.

The level of editing across the strains was moderate to low, with 90% of sites having only 60% or fewer transcriptome reads showing editing; half of transcripts at edited sites are edited 20% or less (Figure 3a). When a site was edited in the transcriptome sequence data from biological replicates from multiple strains, the level of editing was remarkably consistent. For example, we found that at 90% of RNA editing sites, the standard deviation in the level of editing between the replicates within strains was 10% or less, and 15% or less across strains. Based on this observation, we can legitimize the low depth correction that we applied in our analysis and extrapolate that the true number of editing sites, and the level of editing, is remarkably constant across mouse strains (Figures 2a and 3b).

### Strain differences

One striking observation was that the majority of RNA editing sites were shared across many strains: 92% of edited positions were shared amongst five or more strains, and 93% of clustered editing positions were shared amongst nine or more strains (Figure 3b). For the clustered editing positions, if we take into account the correction for low-depth sites (described above), 92% of these positions are edited in 14 or more strains. However, it should be noted that our method has greater power to discover shared edits than singletons because if an edit is initially found in at least one strain, we then go back and genotype all of the other strains for evidence of an edit at that site. Interestingly, although clusters of edits are likely to be shared amongst all strains, individual editing sites both inside and outside of these clusters are often absent in one of the strains (see the drop for 15 strains in Figure 3b). This property appeared to be independent of a particular strain as the same pattern is observed even when single or multiple strains are discarded from the analysis.

The use of Sequenom genotyping for validation confirmed the high conservation of RNA editing sites, with 99% of editing positions being observed in 13 or more strains. When comparing the strain distribution patterns of individual RNA editing sites called by RNA-seq and by Sequenom genotyping, we find good concordance. RNA-seq consistently reported fewer edited sites (that is, RNA editing sites were missed in RNA-seq that were called by Sequenom genotyping), probably because of variable sequence coverage between strains. Indeed, 85% of calls missed in a strain that were subsequently validated by Sequenom genotyping were from sites covered by less than five sequence reads. Less than 2% of all RNA editing sites were specific to one strain only, and most of these were found in the wild-derived strains, with a maximum of 19 private editing sites in PWK/PhJ. When the low depth correction was applied, however, only one private cluster remained for SPRET/EiJ in the 3' UTR of the *Nbeal1 *gene.

We examined the tissue specificity of RNA editing by assaying the set of 611 randomly selected sites described above across a set of 7 tissues (brain, heart, kidney, lung, liver, spleen, thymus) collected from C57BL/6NJ mice. We found that only 61% of sites subject to RNA editing in brain were also edited in the heart transcriptome. Yet lower concordance was observed between the brain and the other tissues (Table 1). This is consistent with earlier estimates [7] and recent studies in human that have shown that brain undergoes significantly higher levels of editing than other tissues [23].

**Table 1:** 

Tissue	Number of edits
Brain	1
Heart	0.61
Kidney	0.6
Lung	0.6
Liver	0.57
Spleen	0.56
Thymus	0.53

### Functional interpretation

**Figure 4:**
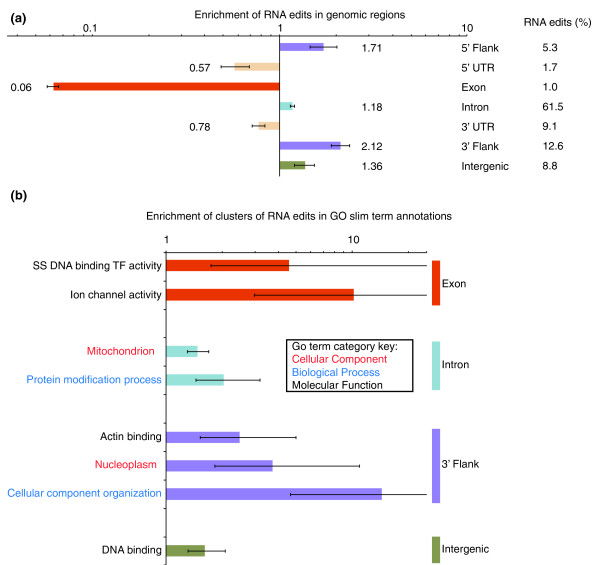
**Depletion/enrichment of RNA editing clusters in **(a) **genic regions and **(b) **associated GO slim terms**. SS, sequence specific; TF, transcription factor.

We investigated whether the overrepresentation of RNA editing sites at sequences outside of the coding regions observed in our study was statistically significant compared to a random distribution generated by placing 50 bp sequence windows containing one or more RNA editing sites on the genome assembly (10,000 iterations, significance level of 0.1%). For this analysis we only considered genomic sequence that was transcribed at a level sufficient to observe RNA editing (depth greater than 10×). The results of this analysis suggested that RNA editing preferentially occurs outside of coding sequence (Figure 4a). We next employed similar simulations using 50 bp windows, but now we exclusively focused on the sequence of expressed coding genes. This analysis demonstrated that three classes of genes (those encoding ion channels or with single-stranded DNA-binding transcription factor activity, or whose product is present in the nucleoplasm) were significantly enriched for RNA editing events (FDR = 0.1%; Figure 4b). When this procedure was replicated for each transcribed genic region in turn (5' or 3' flanking sequence, 5' or 3' untranslated regions, introns, or intergenic sequence) further biases were observed (Figure 4b). Our findings are in contrast to a previous study that identified no significant functional enrichments in human RNA editing sites [24]. In our analysis the terms ion transport, receptor activity, transporter activity and ion channel activity can be attributed to the RNA editing of transcripts from eight genes. Six of these have previously been shown to be substrates for RNA editing (*Gabra3, Gria2, Gria3, Gria4, Grik2 and Kcna1*) and two, *Cacna1d *and *Grik5*, have novel RNA editing sites. We also observed increased editing associated with genes involved in cellular component organization, and to a lesser extent in genes associated with protein modification and DNA binding. It has been shown in human that RNA editing is more prevalent in repetitive elements such as SINEs [25,26]. In our dataset we find a highly significant enrichment bias of RNA editing in SINE and endogenous retrovirus-like elements (Figure s15 in Additional file 2) consistent with the human data.

**Figure 5:**
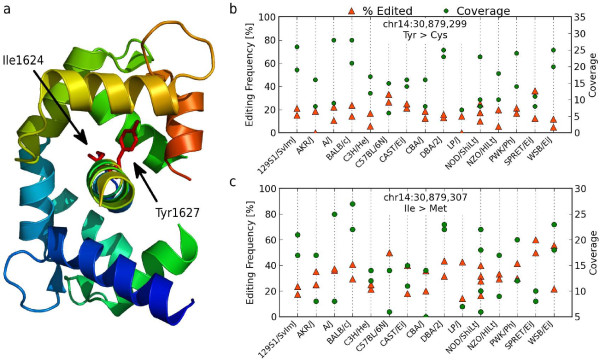
**RNA editing of the *Cacna1d *gene**. **(a) **The *Cacna1d *gene contains two non-synonymous edits in close proximity in the calmodulin binding part of Cav1.2 voltage-gated calcium channel (PDB ID:2BE6). **(b, c) **Editing levels for each strain replicate for the two sites.

As mentioned above the vast majority (87.8%) of the RNA editing we observed was outside of protein coding regions (Figure 4a). In protein coding sequence our call set extends the 23 previously known non-synonymous coding RNA editing sites by a further 30 sites. We validated 24 of these new sites by Sanger sequencing of cDNA (Table 2). We found two interesting novel protein coding edits that we can place on the tertiary structure of *Cacna1d*, which encodes the Cav1.2 voltage-gated calcium channel. These edits are a further source of variation for a protein known to undergo extensive alternative splicing producing multiple isoforms with distinct electrophysiological properties [27]. These two editing sites occur in the calmodulin-binding part of the IQ domain (Figure 5a) and are just 8 bp apart and convert the amino acids Ile1624 and Tyr1627 into Met and Cys, respectively. These two residues and their vicinity are conserved across metazoan species and are known to play an important role in calcium-dependent inactivation and facilitation of the channel [28]. All strains exhibited a moderate but relatively constant level of editing (34% and 16%, respectively) at these sites (Figure 5b, c). Further evidence for the role of RNA editing in generating distinct isoforms of *Cacna1d *was found in the capillary trace data, where we observed transcripts leading to all four possible protein isoforms, with none, both, or either of the sites edited (Figure s11 in Additional file 2).

**Table 2:** 

Chromosome number	Position	Gene	Consequence	Percentage edited	Percentage conserved human	Percentage conserved zebra fish
1	66719288	*Unc80*	S>G	35	100	67
1	75418580	*Speg*	E>G	32	86	43
1	75418639	*Speg*	S>G	81	90	76
12	47801321	*Nova1*	S>G	36	100	0
14	8768555	*Flnb*	S>G	14	95	67
14	8768562	*Flnb*	Q>R	97	81	67
14	30879299	*Cacna1d*	Y>C	16	100	100
14	30879307	*Cacna1d*	I>M	34	100	100
14	76119526	*Cog3*	I>V	23	100	86
16	91655860	*Son*	T>A	17	100	NA
16	91656331	*Son*	T>A	<10	100	NA
16	91656334	*Son*	R>G	47	100	NA
17	27639740	*Grm4*	Q>R	13	100	86
17	45799898	*Tmem63b*	Q>R	68	100	86
18	24118906	*Zfp397*	S>G	12	100	NA
18	24119129	*Zfp397*	Y>C	<10	86	NA
18	24119137	*Zfp397*	S>G	11	86	NA
3	32460393	*Mfn1*	I>V	27	100	95
3	32460397	*Mfn1*	S>L	57	100	95
5	144707220	*Rsph10b2*	R>G	77	NA	NA
6	125190520	*Vamp1*	K>E	47	NA	NA
8	73038612	*2810422J05Rik*	T>A	22	NA	NA
8	73038624	*2810422J05Rik*	I>V	49	NA	NA
9	4456006	*Gria4*	R>G	72	100	76

NA, not available.

As mentioned above, RNA editing within transcribed sequence was relatively constant across strains. There were, however, a few specific instances where one or more strains showed a different editing pattern. In particular, we observed two independent examples where the strain distribution pattern suggested a functional role of RNA editing towards preserving the ancestral genomic sequence state of a transcript despite a divergent genomic sequence change. For example, the 3' UTR of the *Cds2 *gene (CDP-diacylglycerol synthase) contains the site chr2:132,135,391, which is edited in approximately 50% of transcripts from A-to-G in all strains with the exception of three wild-derived strains (CAST/EiJ, PWK/PhJ and SPRET/EiJ), which have the corresponding base change as a genomic change at the site (Figure s12 in Additional file 2). Another example occurs in the 3' UTR of *Tox4 *at chr14:52,913,904, where all strains have an A-to-G SNV with the exception of C57BL/6NJ, which has an A genomic base that is substantially edited to a G (Figure s13 in Additional file 2). Tian *et al*. [29] previously suggested that RNA editing may preserve an evolutionary intermediary that allows the function of a protein to be maintained despite genomic sequence divergence.

## Discussion

We present results for the most comprehensive survey of RNA editing across the mouse genome performed thus far, and one of the largest for any organism. We produced an initial conservative set of RNA editing calls by applying stringent filters to remove systematic alignment artifacts. Using knowledge of the local clustering of A-to-G editing sites, we then extend this call set to 7,389 sites. Our analysis confirms that adenosine deamination is the primary mechanism responsible for RNA editing in the mouse as we find just a small fraction (<0.1%) of edits that are not A-to-I sequence changes. This result is in agreement with a recent analysis of RNA editing within the human transcriptome [12].

We found that for 23% of the RNA editing sites we identified (1,710 out of 7,389), we could not determine the edited strand confidently because of missing annotation or because there were overlapping genes on opposite strands. Validation using Sequenom genotyping, however, confirmed these positions as being true RNA editing sites.

One of the notable features of our editing calls is the striking conservation of editing sites across mouse strains, including the evolutionarily distant wild-derived strains. In particular, we observed that 92% of clustered RNA editing sites are edited in 14 or more strains and for sites shared between strains the editing levels between strain replicates was almost identical to the level between strains (at 90% of sites the standard deviation is 10% within strains and 15% across strains). We believe that variability in the overall editing rate in different strains (Figure 2b) is most likely not attributable to biological differences, but reflects differences in transcriptome sequence coverage (and hence our sensitivity to detect edited bases), alignment issues, including the influence of SNPs and indels close to editing sites, and the influence of other types of systematic errors. Although we did not specifically validate the level of editing calculated from the RNA-seq data, the single molecule counting approach we applied is analogous to those used for Chip-seq and expression analysis where sequencing is the gold standard. Furthermore, RNA-seq was recently shown to provide good concordance with clonal sequence validation of RNA editing sites [12].

The high level of conservation of RNA editing sites and of the rate of editing amongst the mouse strains examined in this study is remarkable considering the level of genomic sequence variation between them. For example, there are more than 35 million single nucleotide differences between SPRET/EiJ and the strain C57BL/6NJ [13], yet the positions of editing in the brain transcriptomes of these strains are overwhelmingly conserved. Importantly, a large proportion of RNA editing sites (90.5%) could not be lifted over to the human genome owing to the fact that most RNA editing takes place in sequence that is repetitive, or is not highly conserved. Interestingly, we found a few instances where RNA editing at one site correlated with genomic base differences at nearby sites. For example, the presence of a SNP in *Cds2 *(chr2:123,135,391) correlated perfectly with the absence of RNA editing at a site 225 bp downstream (chr2:132,135,616); 80% of *Cds2 *transcripts are edited in strains without this SNP (Figure s12 in Additional file 2). It is possible that the reason for this and other differences (Figures s13 and s14 in Additional file 2) is an alteration of the double-stranded RNA structure of the transcript caused by genomic variation. We are not, however, able to confirm this hypothesis since the analysis of the RNA sequence structure of *Cds2 *using software tools, including mFold, was inconclusive.

We identified and validated 24 previously unknown RNA editing sites in protein coding sequence and found most of the known sites (19 out of 23). Of the four known sites we missed, two were poorly covered by RNA-seq data in our study (less than ten reads). We highlight two particularly interesting examples of the effect of RNA editing. In the *Cacna1d *gene we find two proximal non-synonymous coding RNA editing sites that were edited at a relatively constant level across all strains. Another example is an edit in a *Cds2 *transcript that occurred in all strains except the wild-derived strains, which have the corresponding A-to-G base change in their genomic sequence. In rat, the corresponding orthologous position in *Cds2 *is a G and agrees with the wild strains, indicating that the G-to-A SNP in the laboratory strains potentially occurred after their divergence. Thus, RNA editing may act on this site in the laboratory strains to preserve the ancestral sequence of the 3' UTR of the *Cds2 *gene.

## Conclusions

RNA editing is known to be an important biochemical process for the normal functioning of many organisms. In this paper we used RNA-seq of whole brain and the matched genome sequences of 15 mouse strains to generate a comprehensive picture of RNA editing in this tissue. We found that individual editing sites and the level of editing at these sites are highly conserved despite millions of years of evolution. Further, our analysis showed that RNA editing sites are depleted in coding regions of the genome but enriched in transcripts from ion channels, DNA-binding transcription factors, and the transcripts of genes associated with the nucleoplasm. We also provide further evidence for a previously proposed mechanism whereby RNA editing is involved in maintaining the functional state of a protein despite evolutionary sequence divergence. Our study extends the list of known coding edits significantly, and in the *Cacna1d *gene, we find two RNA editing positions that we predict will function to expand the repertoire of protein products generated by this gene.

## Materials and methods

### Samples and sequencing

The 15 inbred laboratory mouse strains (Figure 2) were sequenced on the Illumina GAIIx platform to an average of 25× mapped depth [13]. We generated between 5.2 and 21.2 Gbp of RNA-seq data per sample on the Illumina GAIIx platform with paired-end reads 76 bp in length from whole-brain tissue with a minimum of two biological replicates per strain [13]. All mice/samples were adults, 8 to 12 weeks old.

### Mapping and variant calling

We aligned the RNA-seq reads to the MM9/NCBIM37 reference genome with BWA v0.5.5 [29] and initially called SNVs with samtools/bcftools v0.1.16 (r963:234) [15] (Figure s5 in Additional file 2). The reads from candidate sites were realigned in a splice-aware manner using exonerate v2.4.0 with parameters: -m e2g -n 2 -Q dna -T dna --wordjump 20 -s 300 --fsmmemory 1024 --saturatethreshold 100 [16]. The End Distance Bias and Strand Bias filters of samtools/bcftools were set to 0.05 and 0.01. While End Distance Bias checks if variant bases tend to occur at a fixed distance from the end of reads using a *t*-test, the Variant Distance Bias evaluates the likelihood of the mean pairwise distance of the variant bases in the aligned portion of the reads. The Variant Distance Bias filter (Figure s4 in Additional file 2) was set to 0.015 and this filtering method was contributed to the main publicly available development branch of samtools/bcftools.

### Low depth correction

If there was RNA editing in one or more strains (say N% of reads are edited), we test the hypothesis that we are not seeing editing in another strain because of low sequencing depth. We assume that either the site is edited to the same extent as in the other strains or it is not edited at all. This is an approximation justified because when a site is subject to RNA editing, the level of editing does not vary much across the strains and biological replicates (Figure 3a). We then use a one-sided binomial test to reject the hypothesis that low coverage is the reason for not observing editing at a site: with K reads covering the site we calculate the probability of obtaining this number of non-edited reads under the assumption that the site is edited: (N × 0.01)^K^, where N is the percentage of reads edited in other strains. If the probability is below the significance threshold (0.05), we reject the hypothesis that low coverage is the reason for not observing the edit. Otherwise, we say that the site may be edited.

### Testing the distribution of editing clusters

To test the distribution of clusters of RNA editing across genic regions and Gene Ontology (GO) annotations, we used the Genomic Association Tester (GAT) algorithm [30]. The clusters were defined as consecutive editing sites with distances smaller than 50 bp.

We divided the mouse genome into genic regions with respect to coding genes with orthologues in humans in order to discriminate against false gene predictions. Genic regions were defined as 5' flanking and 3' flanking sequence (5 kb upstream and downstream of genes, respectively), intergenic space, 5' UTRs, 3' UTRs, introns and exons. We tested the density of RNA edit clusters across these genic regions to a random distribution obtained using simulations. Next, we annotated the genic regions according to their associated GO slim terms [31] from Ensembl mart version 60 [32] and compared the density of RNA editing clusters within regions associated with GO slim terms with densities obtained from random simulations.

GAT calculates the probability of an observed density compared to an expected distribution through 10,000 randomized permutations of input data. To simulate the random null expectation, permutations are performed taking into account potential biases in chromosome, genic region and isochore densities. Multiple testing corrections were applied with the Benjamini-Hochberg method [33] for a stringent FDR of 0.1%.

### Validation

Genotyping was performed using the iPLEX™ Gold Assay (Sequenom^® ^Inc. San Diego, CA, USA). Assays for all SNPs and SNVs were designed using the eXTEND suite and MassARRAY Assay Design software version 3.1 (Sequenom^® ^Inc.). Amplification was performed in a total volume of 5 μl containing approximately 10 ng genomic DNA, 100 nM of each PCR primer, 500 μM of each dNTP, 1.25 × PCR buffer (Qiagen, Germantown, MD, USA), 1.625 mM MgCl_2 _and 1 U HotStar Taq^® ^(Qiagen). Reactions were heated to 94°C for 15 minutes followed by 45 cycles at 94°C for 20 s, 56°C for 30 s and 72°C for 1 minute, then a final extension at 72°C for 3 minutes. Unincorporated dNTPs were SAP *(*Shrimp Alkaline Phosphatase) digested prior to iPLEX™ Gold allele specific extension with mass-modified ddNTPs using an iPLEX Gold reagent kit (Sequenom^® ^Inc.). SAP digestion and extension were performed according to the manufacturer's instructions with reaction extension primer concentrations adjusted to between 0.7 and 1.8 μM, dependent upon primer mass. Extension products were desalted and dispensed onto a SpectroCHIP using a MassARRAY Nanodispenser prior to MALDI-TOF (matrix-assisted laser desorption/ionization-time of flight) this is fine analysis with a MassARRAY Analyzer Compact mass spectrometer. Genotypes were automatically assigned and manually confirmed using MassARRAY TyperAnalyzer software version 4.0 (Sequenom^® ^Inc.).

To validate clustered RNA editing sites, we used PCR to amplify cDNA sequences generated from brain tissue obtained from C57BL/6J, 129S1/SvImJ, WSB/EiJ and PWK/PhJ mice. Primers were designed to flank the edited positions. PCR products were excised from a 3% agarose gel, purified using the QIAquick Gel Extraction Kit (Qiagen) and ligated into the pGEM-T Easy Vector (Promega, Southampton, UK) for cloning, according to the manufacturers' instructions. Multiple clones from each ligation (at least 12) were sequenced using Sanger-based capillary sequencing. Traces and primers are available from our ftp site [34].

### Accession numbers

The RNA-seq reads have been submitted to the European Nucleotide Archive under accession number [ERP000614].

## Abbreviations

ADAR: adenosine deaminase acting on RNA; bp: base pair; FDR: false discovery rate; GAT: Genomic Association Tester; GO: Gene Ontology; SINE: short interspersed element; SNV: single nucleotide variant; UTR: untranslated region.

## Competing interests

The authors declare that they have no competing interests.

## Authors' contributions

PD, RD, JF, TMK and DJA conceived the ideas for the study. PD and TMK carried out the computational analysis. RM, JB, and SB carried out the wet lab validation experiments. CN and CP carried out the functional classes and GO categories analysis of the calls. PD, TMK, CN, and DJA wrote the manuscript, which was approved by all of the authors.

## Supplementary Material

Additional file 1 **Release-v1.vcf.gz is a VCF file listing the RNA-editing sites and their relative strain distribution patterns**. Click here for file

Additional file 2 **Supplementary figures**. Click here for file

Additional file 3 **Supplementary Table s1 with the sites validated on the Sequenom platform**. Click here for file
