# Supplementary material for: High levels of RNA-editing site conservation amongst 15 laboratory mouse strains
Source: Genome Biol. 2012 Apr 23;13(4):r26. doi: 10.1186/gb-2012-13-4-r26 (PMC3446300; doi:10.1186/gb-2012-13-4-r26)
Supplement: Additional file 2 — Supplementary figures. [file gb-2012-13-4-r26-S2.PPT]

## Slide 1
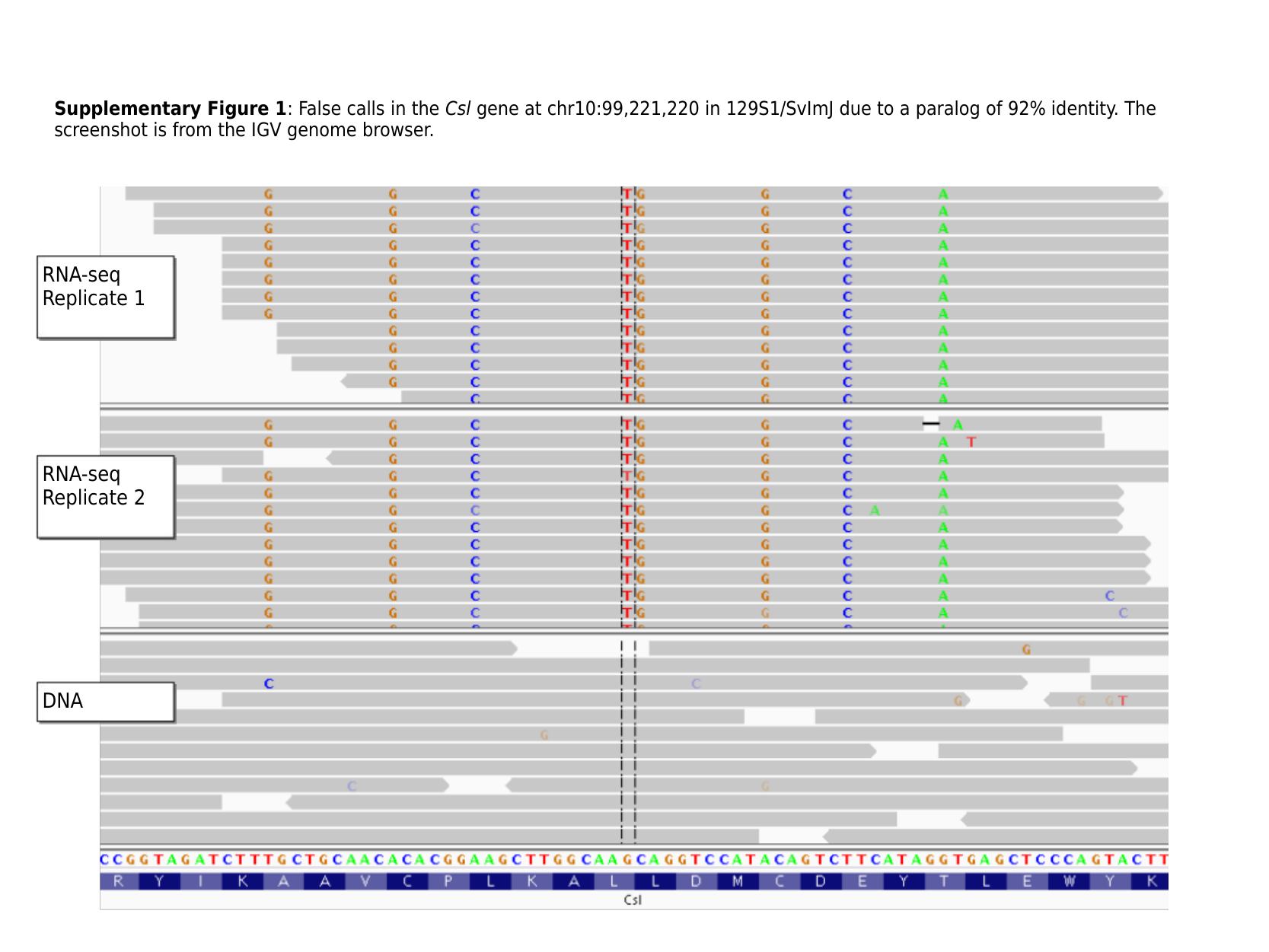

Supplementary Figure 1: False calls in the Csl gene at chr10:99,221,220 in 129S1/SvImJ due to a paralog of 92% identity. The screenshot is from the IGV genome browser.
RNA-seq
Replicate 1
RNA-seq
Replicate 2
DNA

## Slide 2
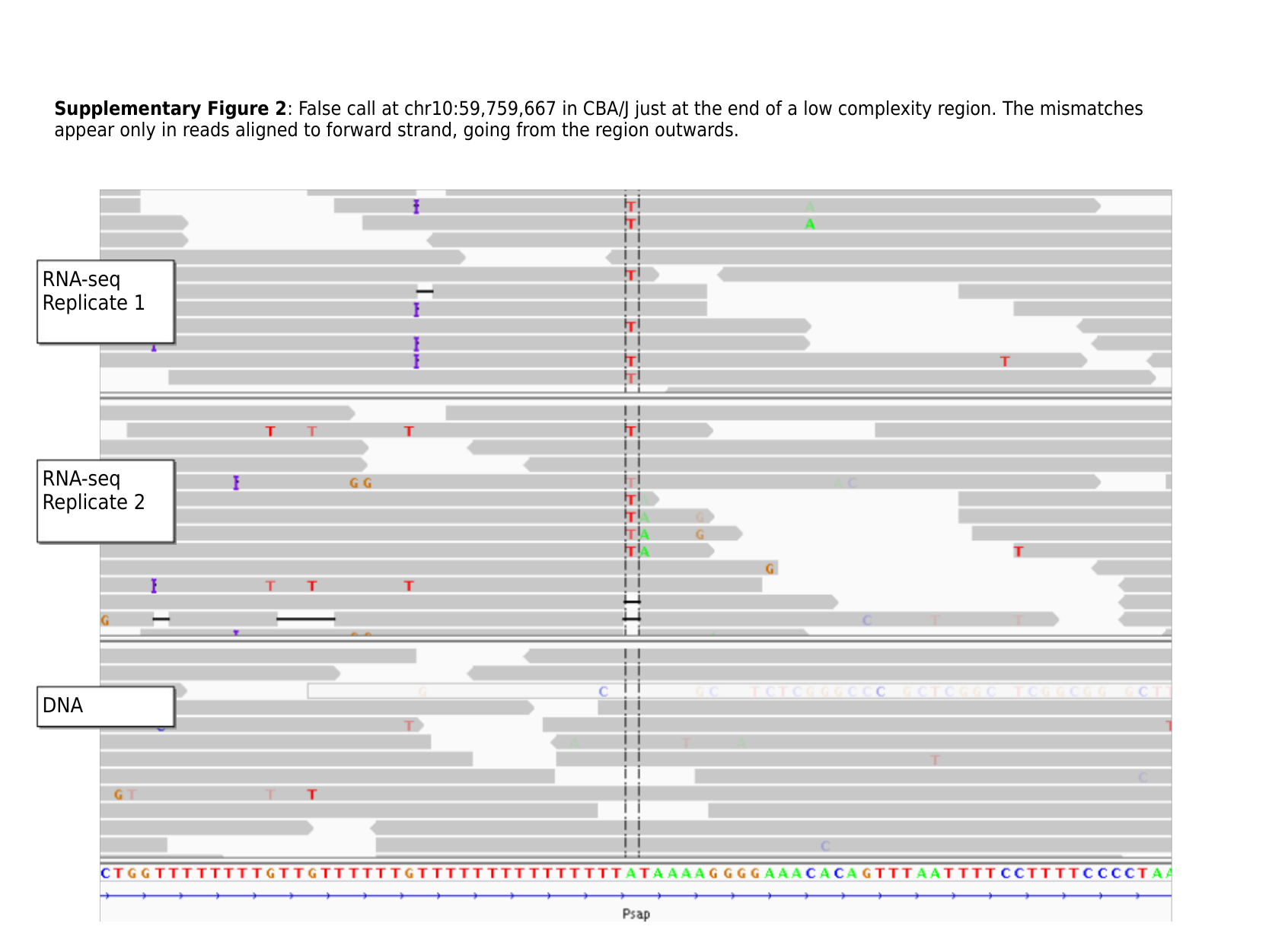

Supplementary Figure 2: False call at chr10:59,759,667 in CBA/J just at the end of a low complexity region. The mismatches appear only in reads aligned to forward strand, going from the region outwards.
RNA-seq
Replicate 1
RNA-seq
Replicate 2
DNA

## Slide 3
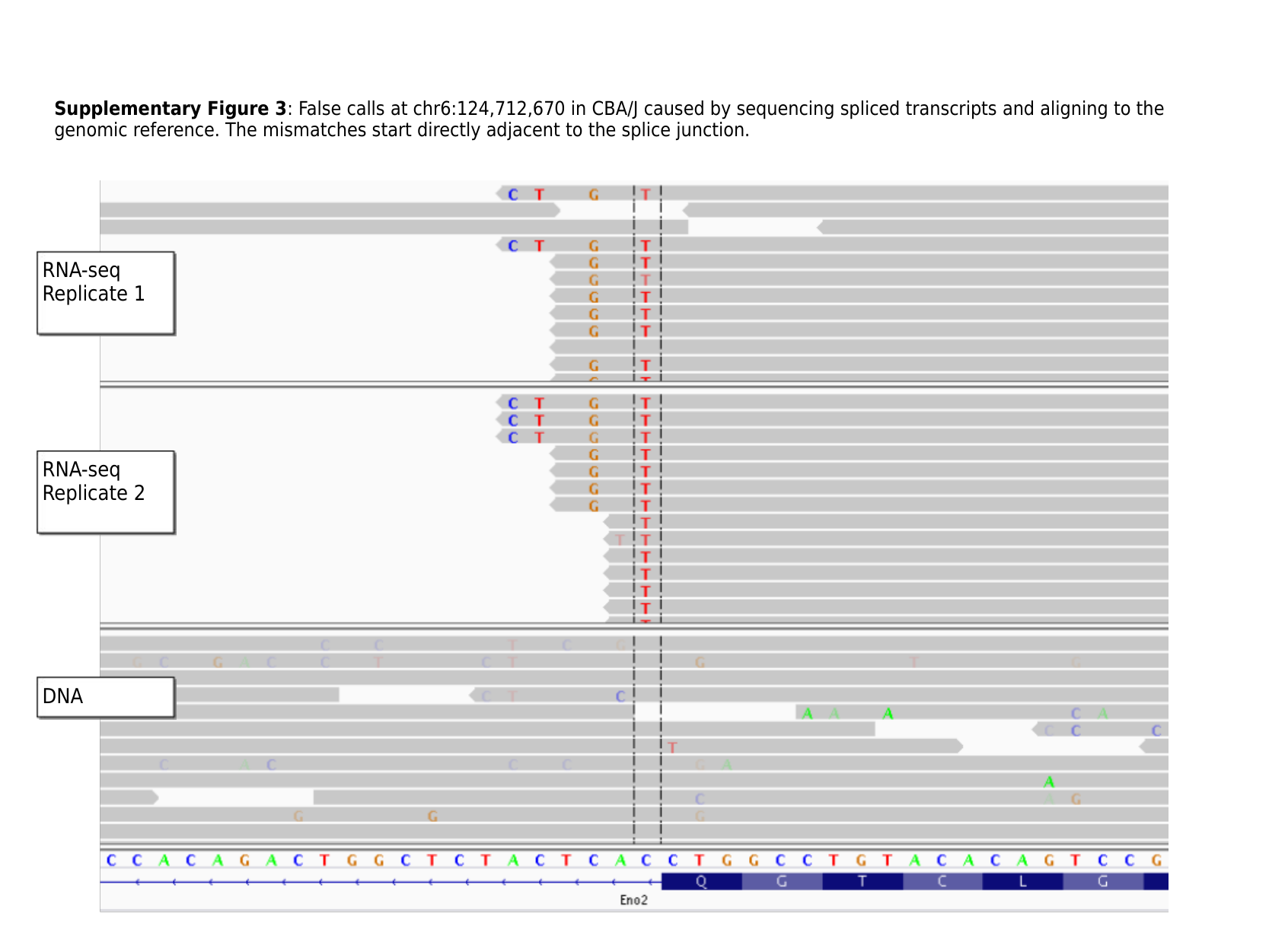

Supplementary Figure 3: False calls at chr6:124,712,670 in CBA/J caused by sequencing spliced transcripts and aligning to the genomic reference. The mismatches start directly adjacent to the splice junction.
RNA-seq
Replicate 1
RNA-seq
Replicate 2
DNA

## Slide 4
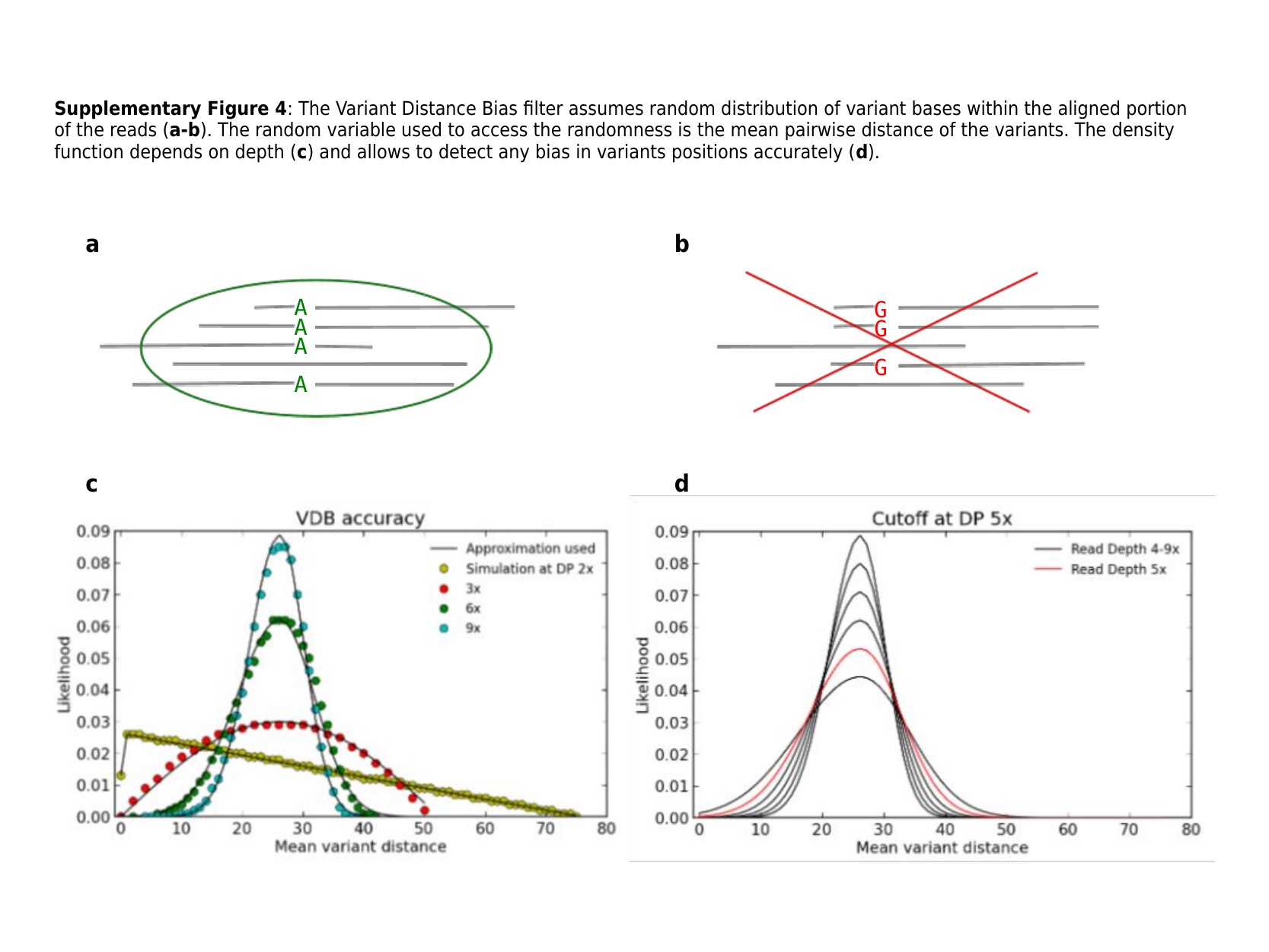

Supplementary Figure 4: The Variant Distance Bias filter assumes random distribution of variant bases within the aligned portion of the reads (a-b). The random variable used to access the randomness is the mean pairwise distance of the variants. The density function depends on depth (c) and allows to detect any bias in variants positions accurately (d).
a
b
A
G
A
G
A
G
A
c
d

## Slide 5
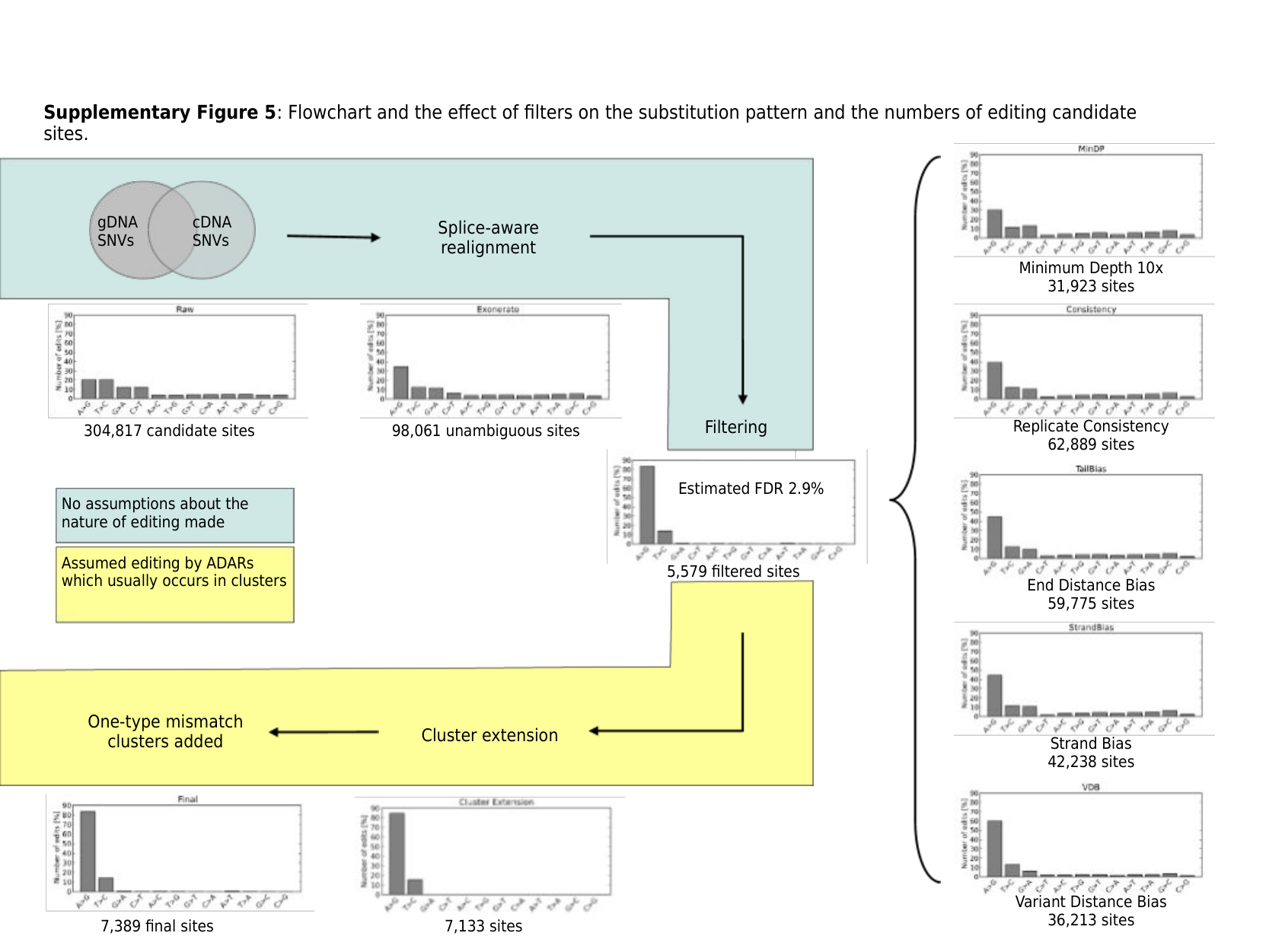

Supplementary Figure 5: Flowchart and the effect of filters on the substitution pattern and the numbers of editing candidate sites.
gDNA
SNVs
cDNA
SNVs
Splice-aware realignment
Minimum Depth 10x
31,923 sites
Filtering
Replicate Consistency
62,889 sites
304,817 candidate sites
98,061 unambiguous sites
Estimated FDR 2.9%
No assumptions about the nature of editing made
Assumed editing by ADARs which usually occurs in clusters
5,579 filtered sites
End Distance Bias
59,775 sites
One-type mismatch clusters added
Cluster extension
Strand Bias
42,238 sites
Variant Distance Bias
36,213 sites
7,389 final sites
7,133 sites

## Slide 6
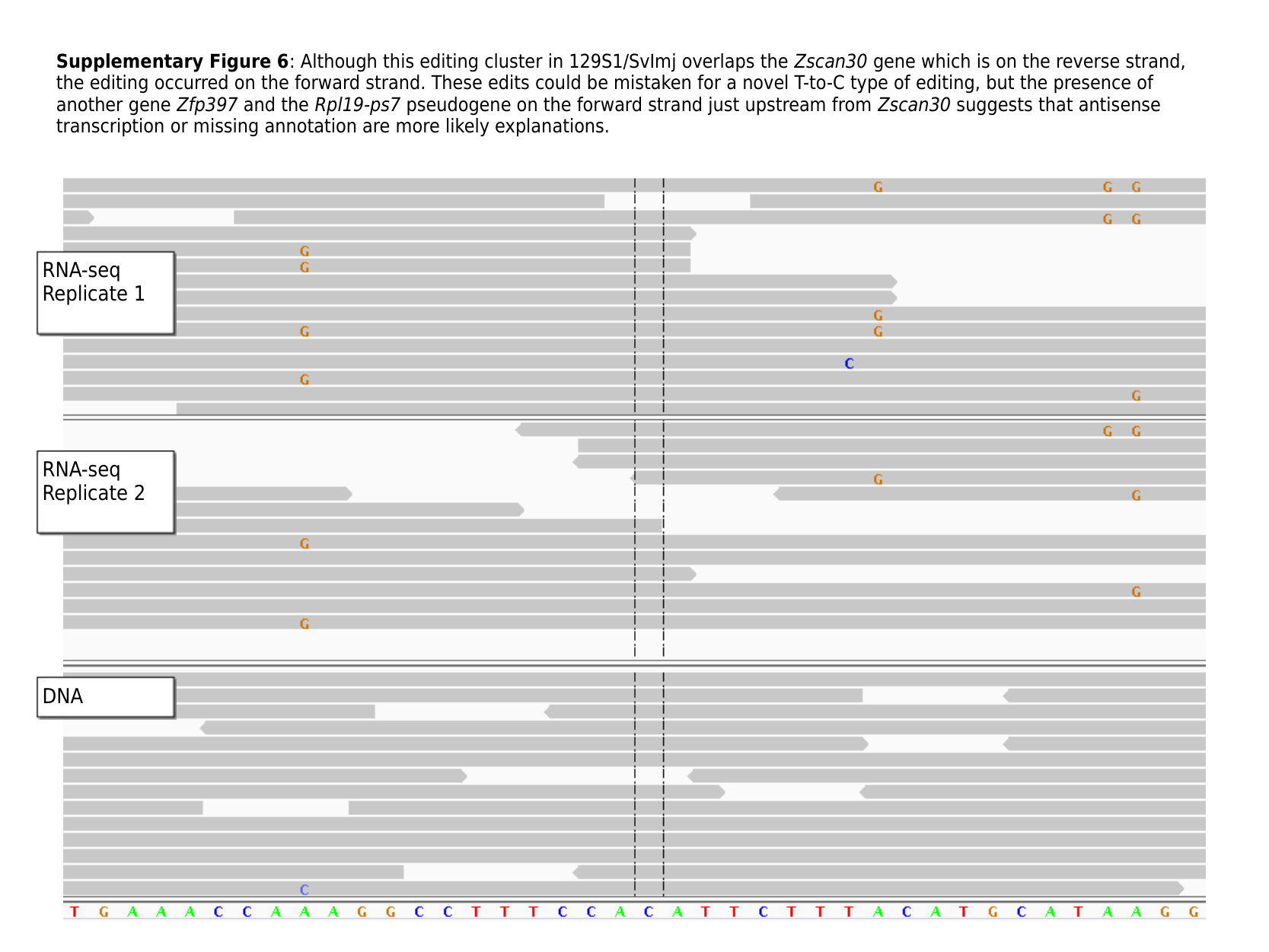

Supplementary Figure 6: Although this editing cluster in 129S1/SvImj overlaps the Zscan30 gene which is on the reverse strand, the editing occurred on the forward strand. These edits could be mistaken for a novel T-to-C type of editing, but the presence of another gene Zfp397 and the Rpl19-ps7 pseudogene on the forward strand just upstream from Zscan30 suggests that antisense transcription or missing annotation are more likely explanations.
RNA-seq
Replicate 1
RNA-seq
Replicate 2
DNA

## Slide 7
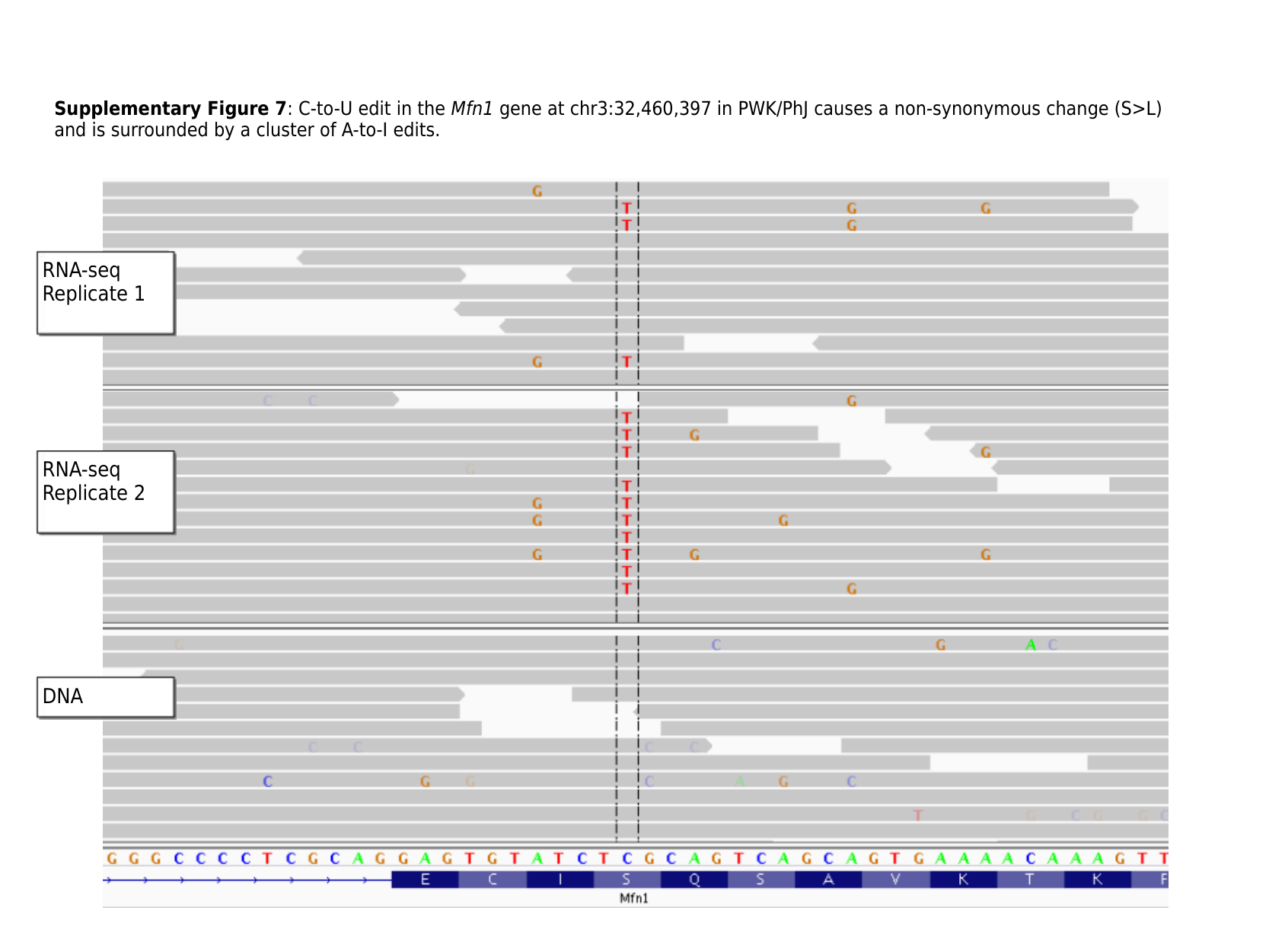

Supplementary Figure 7: C-to-U edit in the Mfn1 gene at chr3:32,460,397 in PWK/PhJ causes a non-synonymous change (S>L) and is surrounded by a cluster of A-to-I edits.
RNA-seq
Replicate 1
RNA-seq
Replicate 2
DNA

## Slide 8
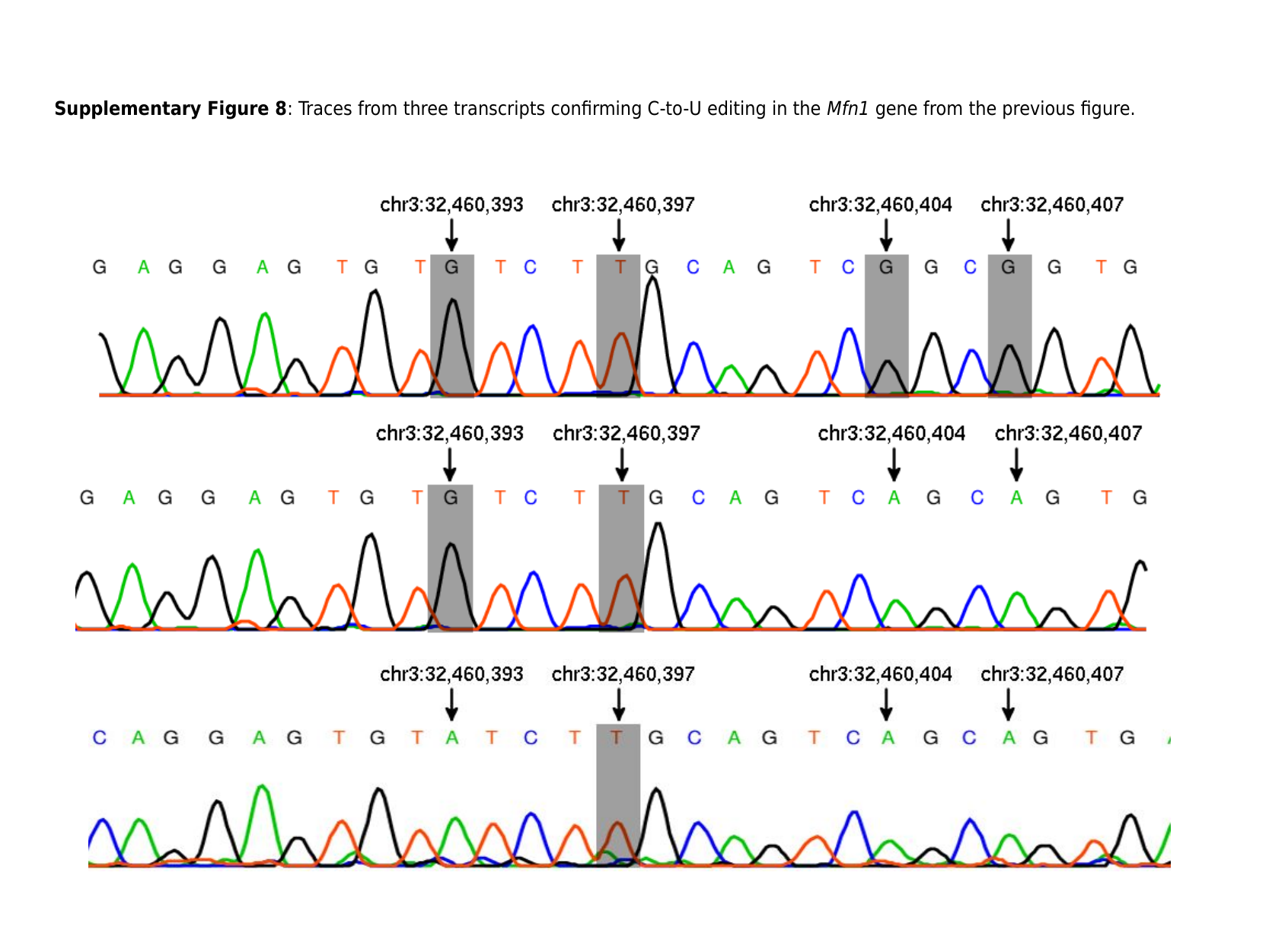

Supplementary Figure 8: Traces from three transcripts confirming C-to-U editing in the Mfn1 gene from the previous figure.

## Slide 9
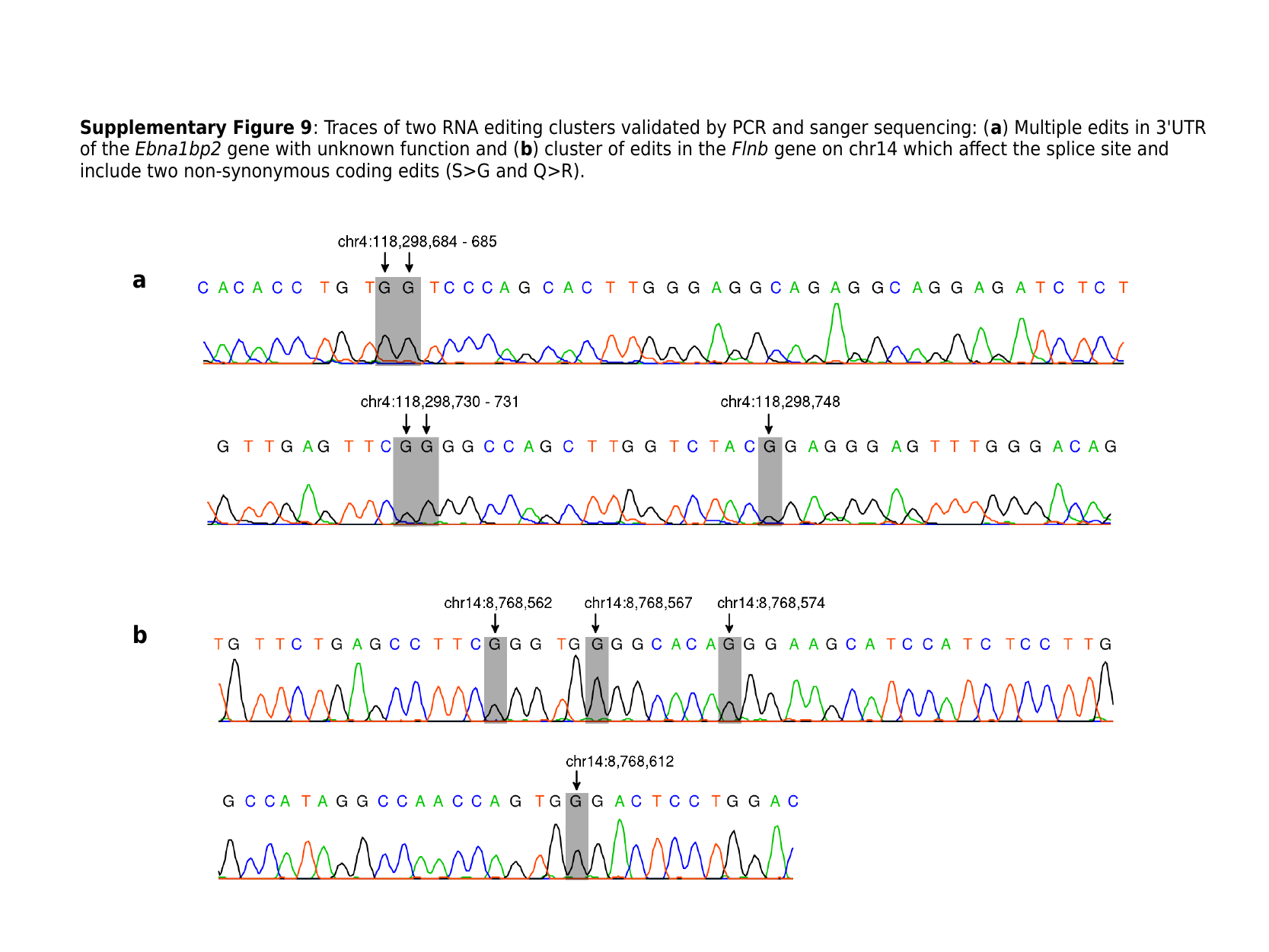

Supplementary Figure 9: Traces of two RNA editing clusters validated by PCR and sanger sequencing: (a) Multiple edits in 3'UTR of the Ebna1bp2 gene with unknown function and (b) cluster of edits in the Flnb gene on chr14 which affect the splice site and include two non-synonymous coding edits (S>G and Q>R).
a
b

## Slide 10
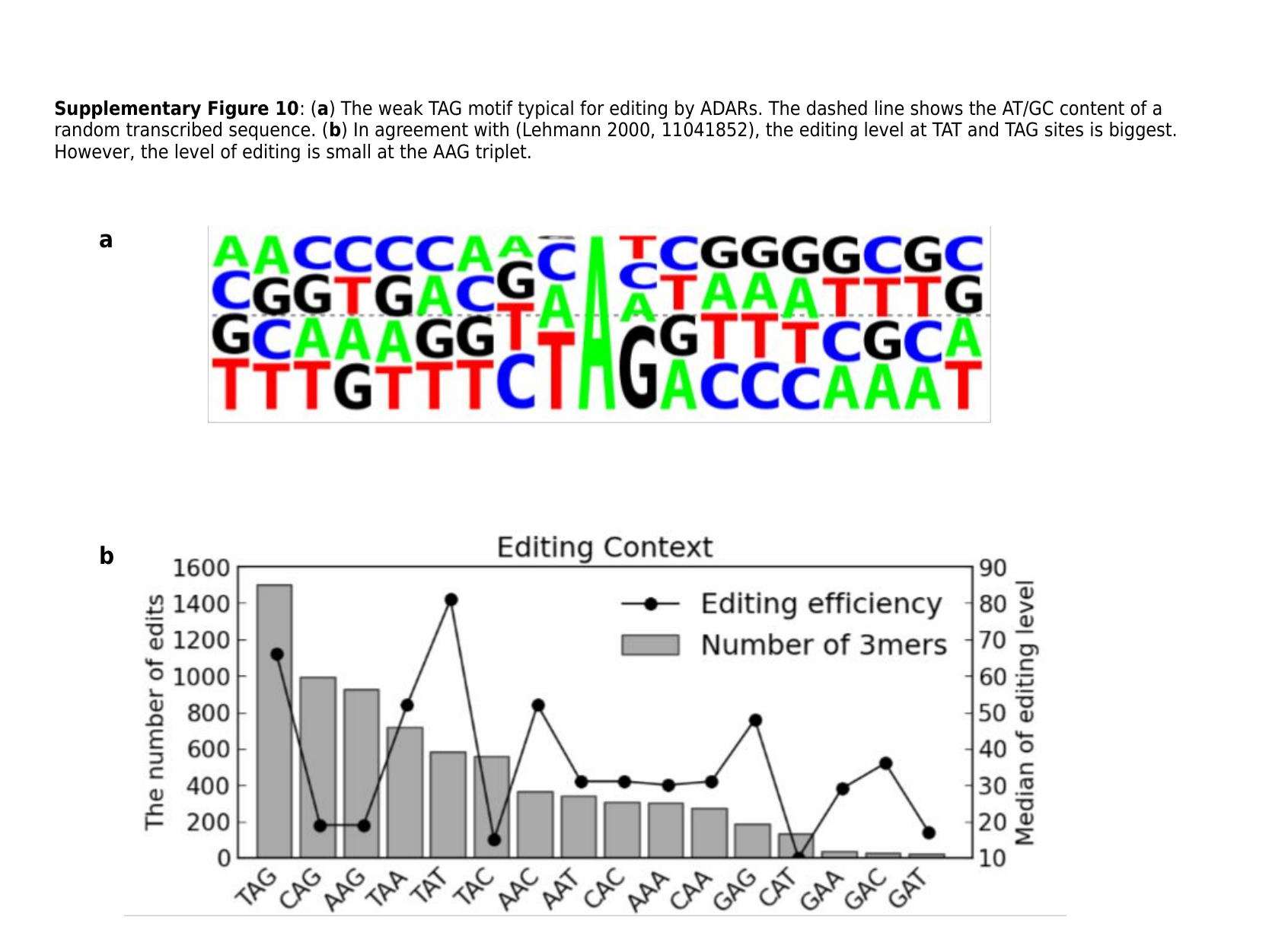

Supplementary Figure 10: (a) The weak TAG motif typical for editing by ADARs. The dashed line shows the AT/GC content of a random transcribed sequence. (b) In agreement with (Lehmann 2000, 11041852), the editing level at TAT and TAG sites is biggest. However, the level of editing is small at the AAG triplet.
a
b

## Slide 11
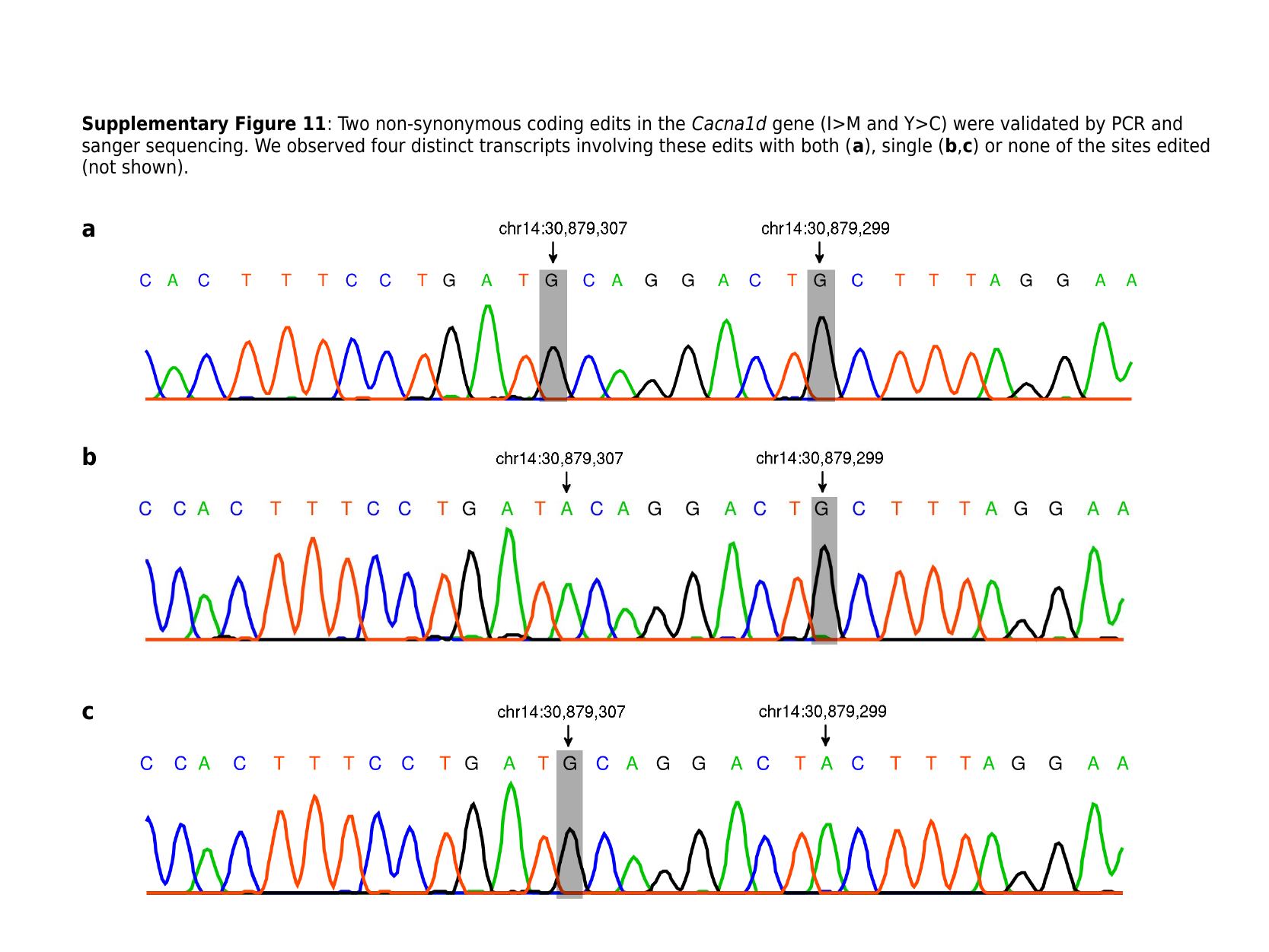

Supplementary Figure 11: Two non-synonymous coding edits in the Cacna1d gene (I>M and Y>C) were validated by PCR and sanger sequencing. We observed four distinct transcripts involving these edits with both (a), single (b,c) or none of the sites edited (not shown).
a
b
c

## Slide 12
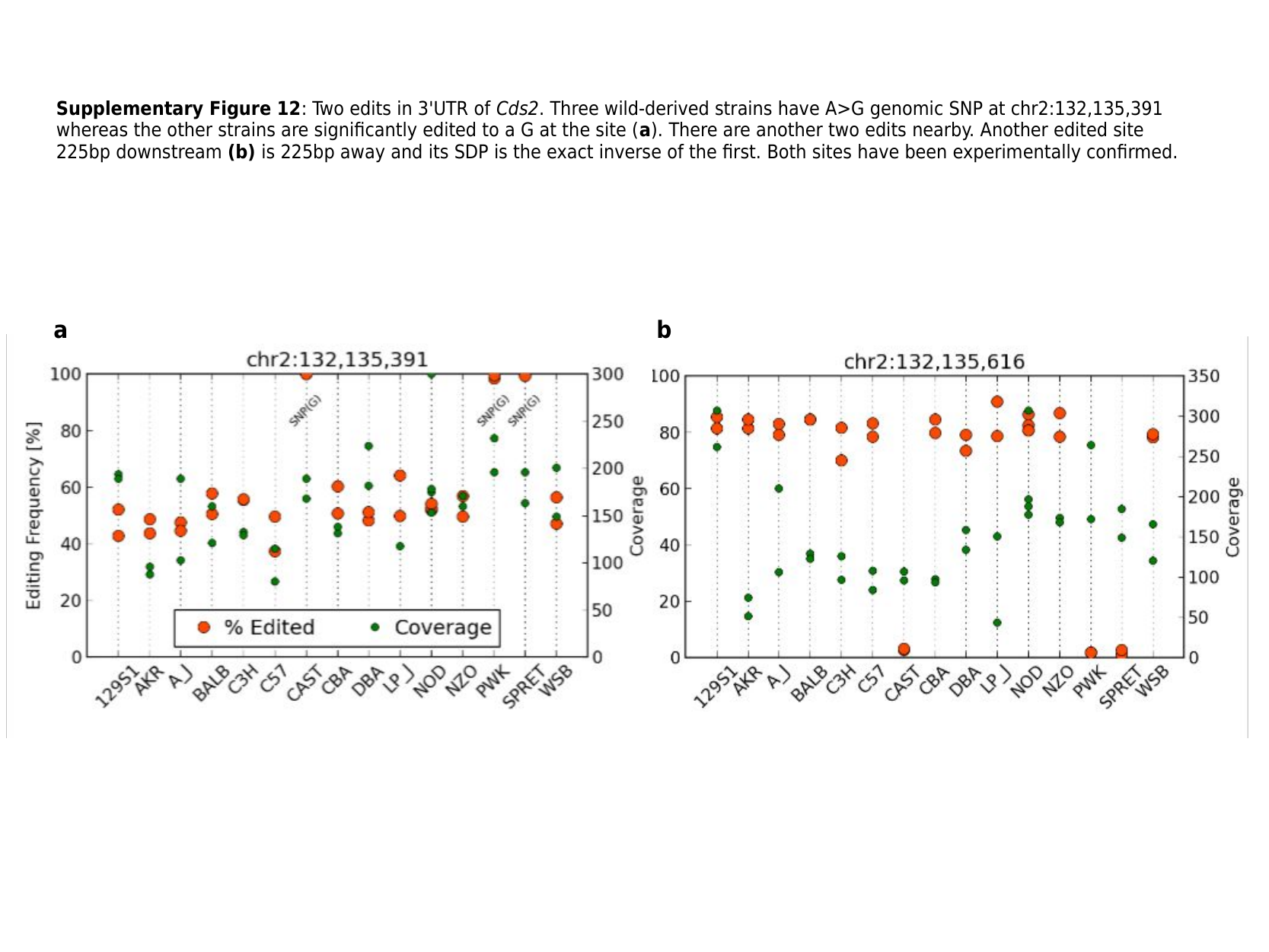

Supplementary Figure 12: Two edits in 3'UTR of Cds2. Three wild-derived strains have A>G genomic SNP at chr2:132,135,391 whereas the other strains are significantly edited to a G at the site (a). There are another two edits nearby. Another edited site 225bp downstream (b) is 225bp away and its SDP is the exact inverse of the first. Both sites have been experimentally confirmed.
a
b

## Slide 13
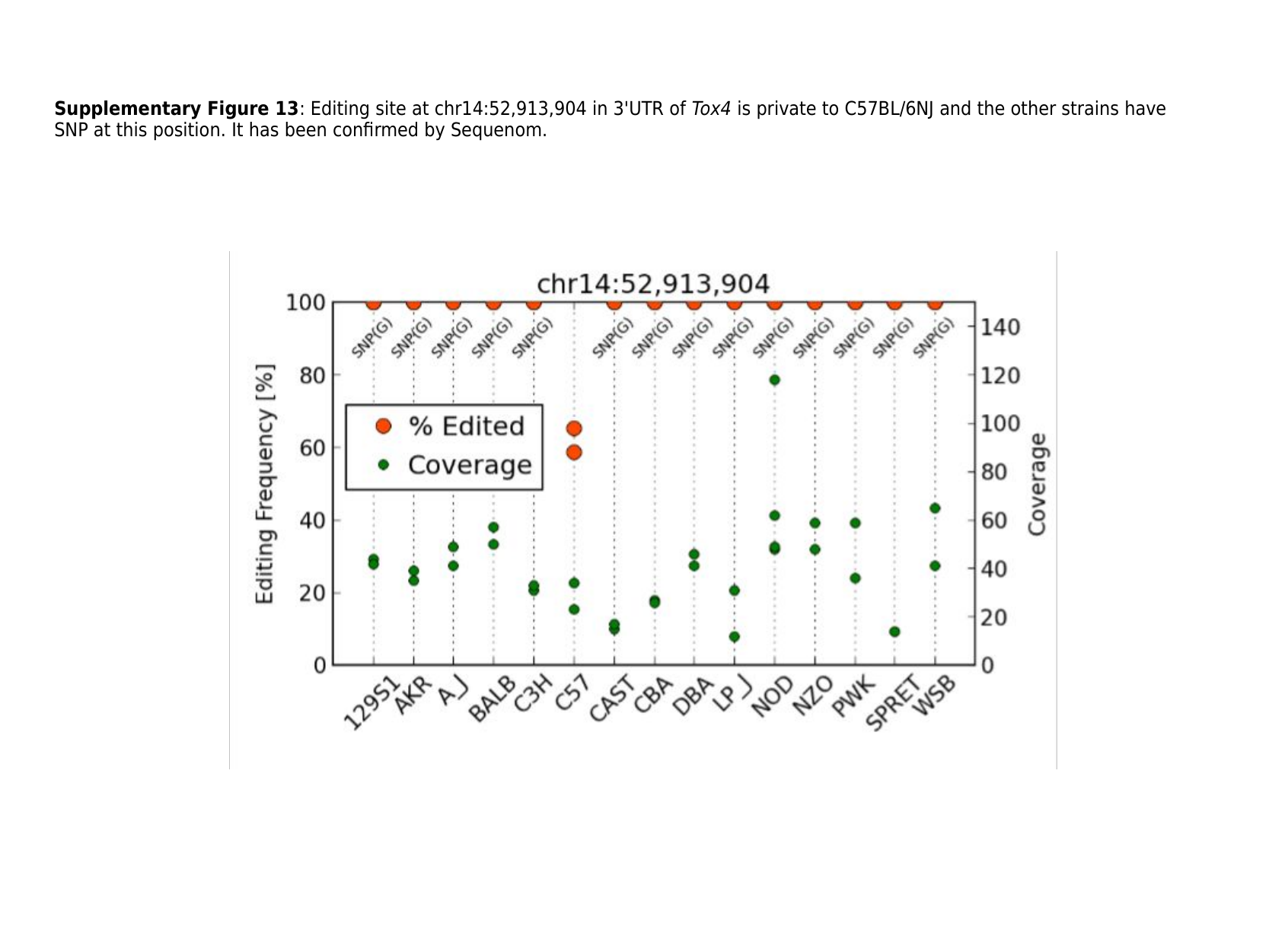

Supplementary Figure 13: Editing site at chr14:52,913,904 in 3'UTR of Tox4 is private to C57BL/6NJ and the other strains have SNP at this position. It has been confirmed by Sequenom.

## Slide 14
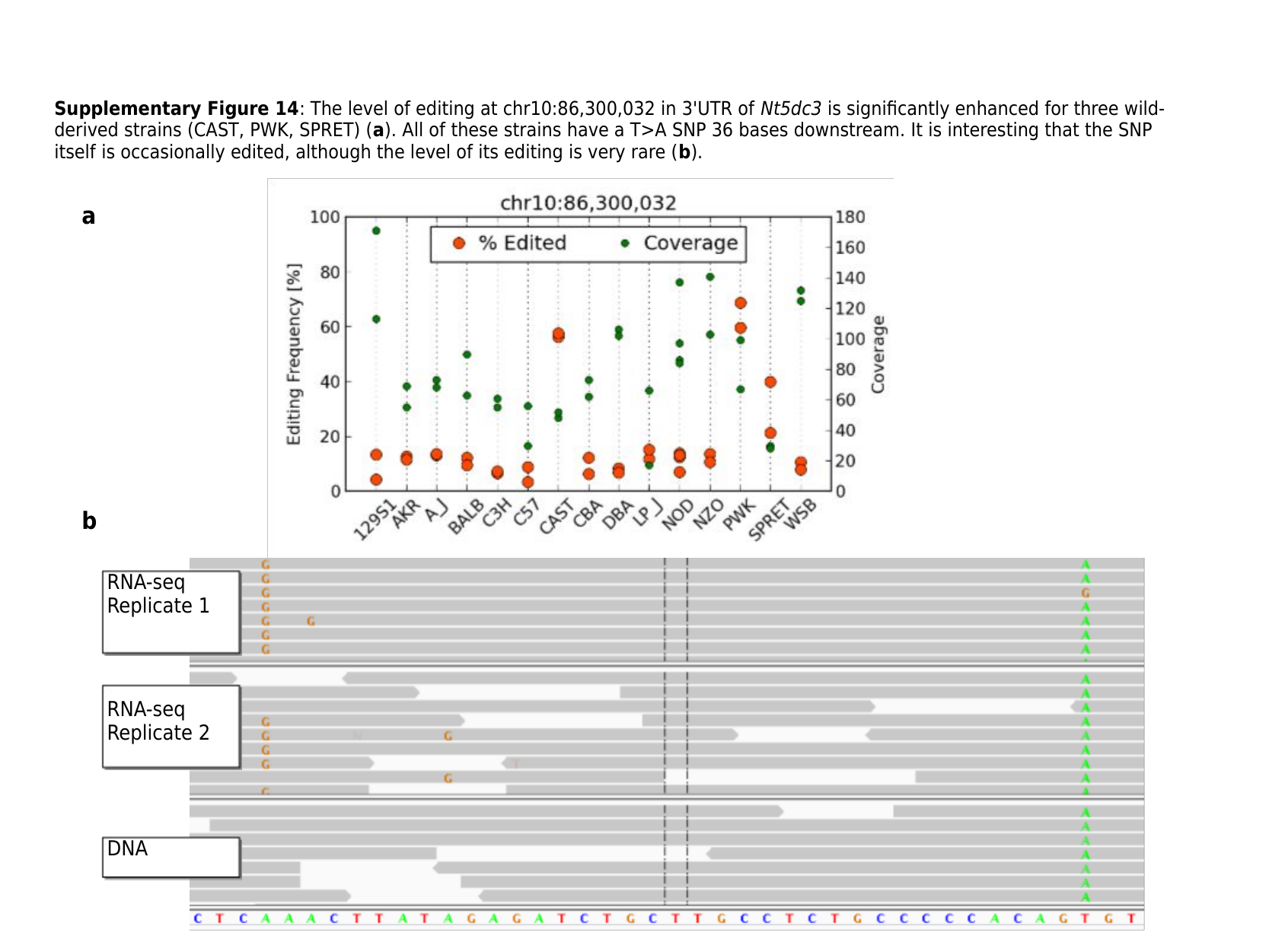

Supplementary Figure 14: The level of editing at chr10:86,300,032 in 3'UTR of Nt5dc3 is significantly enhanced for three wild-derived strains (CAST, PWK, SPRET) (a). All of these strains have a T>A SNP 36 bases downstream. It is interesting that the SNP itself is occasionally edited, although the level of its editing is very rare (b).
a
b
RNA-seq
Replicate 1
RNA-seq
Replicate 2
DNA

## Slide 15
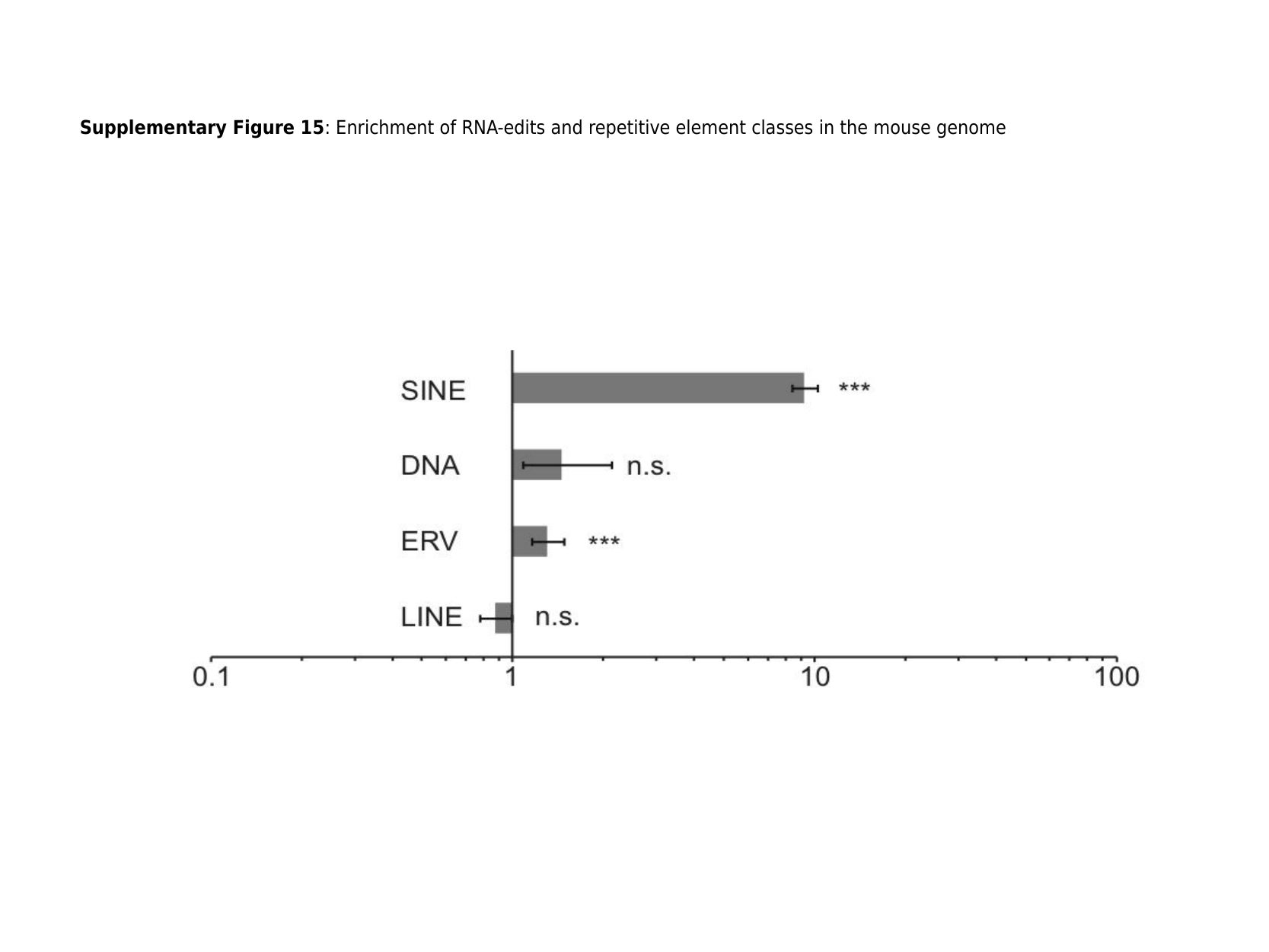

Supplementary Figure 15: Enrichment of RNA-edits and repetitive element classes in the mouse genome
